# Bovine neutrophils kill the sexually-transmitted parasite *Tritrichomonas foetus* using trogocytosis

**DOI:** 10.1007/s11259-023-10260-5

**Published:** 2023-11-16

**Authors:** Jonathan Najera, Michael M. Berry, Ashley D. Ramirez, Bryan Ramirez Reyes, Arielle Angel, Juanita K. Jellyman, Frances Mercer

**Affiliations:** Department of Biological Sciences, California State Polytechnic University Pomona, Pomona, CA 91768 USA

**Keywords:** *Tritrichomonas foetus*, Trichomonosis, Trichomoniasis, Neutrophil, Trogocytosis

## Abstract

The protozoan parasite *Tritrichomonas foetus* (*T. foetus*) is the causative organism of bovine trichomonosis (also referred to as trichomoniasis), a sexually-transmitted infection that reduces fertility in cattle. Efforts to control trichomonosis on cattle farms are hindered by the discouragement of antibiotic use in agriculture, and the incomplete, short-lived protection conferred by the current vaccines. A more complete mechanistic understanding of what effective immunity to *T. foetus* entails could enable the development of more robust infection control strategies. While neutrophils, the primary responders to infection, are present in infected tissues and have been shown to kill the parasite in vitro, the mechanism they use for parasite killing has not been established. Here, we show that primary bovine neutrophils isolated from peripheral blood rapidly kill *T. foetus *in vitro in a dose-dependent manner, and that optimal parasite killing is reduced by inhibitors of trogocytosis. We also use imaging to show that bovine neutrophils surround *T. foetus* and trogocytose its membrane. These findings are consistent with killing via trogocytosis, a recently described novel neutrophil antimicrobial mechanism.

**Supplementary Information:**

The online version contains supplementary material available at 10.1007/s11259-023-10260-5.

## Introduction

Bovine trichomonosis is a sexually-transmitted infection with global distribution. The infection reduces fertility and increases spontaneous abortion rates in infected cows, thereby contributing to a reduction in the births of viable calves in affected herds (BonDurant 1997, Rhyan et al. 1995). The standard line of treatment for human trichomonosis is the imidazole family of antibiotics, however the use of these is prohibited in agriculture in the United States (Love et al. 2017). Therefore, separation of cows from bulls with fencing, testing and slaughter of infected bulls, and artificial insemination are recommended to reduce the incidence of trichomonosis on farms. In the United States, a patchwork of state regulations has resulted in under-reporting of the infection, but infected bulls are more common in western states, particularly California and Texas, where free-ranging is used (Martin et al. 2021). A whole-cell inactivated vaccine called TrichGuard™ exists for bovine trichomonosis, requiring two annual boosters before each breeding season. Vaccines have been reported to induce anti-*T.foetus* antibodies, and increase conception rates, (Corbeil et al. 2003, 2001; Kvasnicka et al. 1992, Ortega-Mora et al. 2022). However, they do not completely prevent infection in vaccinated individuals, confer long-term immunological memory, or completely prevent abortion (Edmondson et al. 2017). Overall, more research into the correlates of protection for bovine trichinosis is needed (Baltzell et al. 2013). A more complete understanding of cellular and molecular immunity against bovine trichomonosis could enable the rational design of more robust vaccines and treatments for the infection.

The causative agent of bovine trichomonosis is the parasite *Tritrichomonas foetus,* which is named for its effects on fetal weight and mortality (Rae and Crews 2006). *T. foetus* is a unicellular, flagellated protozoan parasite. It infects the prepuce in males, producing no symptoms or pathology, and does not get cleared. Therefore, bulls are long-term asymptomatic carriers (Rae and Crews 2006). In females, it infects the vagina, travelling up the cervix, and colonizing the uterus within two weeks (Parsonson et al. 1976, Singh et al. 2004). Unlike males, infected females usually clear the infection naturally. However, there is considerable variability in the timing and efficiency of clearance from cow-to-cow. Furthermore, even in cows that can clear the parasite, no long-term immunity is formed: cows may become infected again during the next breeding season (Collántes-Fernández et al. 2018; Rae and Crews 2006).

Immune responses against *T. foetus* have been extensively characterized at the organismal level, and cattle are known to make antibodies against *T. foetus* (BonDurant et al. 1993; Corbeil et al. 1998, 2001; Ikeda et al. 1995; Voyich et al. 2001). Moreover, neutrophils, also known as Polymorphonuclear Cells (PMNs), are abundantly recruited to the site of infection (Corbeil et al. 2003), and have been shown to kill *T. foetus *in vitro (Aydintug et al. 1993). However, PMNs have four known modes by which they can kill targets (Deniset and Kubes 2016; Kolaczkowska and Kubes 2013; Matlung et al. 2018; Mercer et al. 2018), and the specific mechanism that PMNs use to kill *T. foetus* is not known.

We have recently shown that human PMNs rapidly kill the human-infective trichomonad parasite, *Trichomonas vaginalis*, using a novel PMN antimicrobial mechanism called trogocytosis (Mercer et al. 2018). Trogocytosis is a cellular phenomenon in which plasma membrane is transferred from a donor cell to a recipient cell in a cell–cell contact-dependent manner, and has been increasingly observed to occur in more and more cell types, ranging from immune cells, to protozoans, to embryonic cells (Bettadapur et al. 2020; Uribe-Querol and Rosales 2021). While some forms of trogocytosis involve swapping of membrane proteins between target and recipient cells (i.e. cellular gnawing) (Ochs et al. 2023; Samer et al. 2023; Schriek and Villadangos 2023), other forms of trogocytosis involve engulfment of trogocytosed material by the recipient cell (i.e. nibbling) (Bettadapur et al. 2020; Gilmartin et al. 2017; Ralston et al. 2014; Steele et al. 2016). It is unclear whether just membrane, or additional cytosolic components and organelles, can be trogocytosed by neutrophils from *T. vaginalis* (Mercer and Johnson 2018). Furthermore, while we have previously shown that at least some membrane fragments of *T. vaginalis* do become internalized by neutrophils following trogocytosis (Mercer et al. 2018), the relative amount of “gnawed” versus “nibbled” parasite material, and which process causes more damage to the parasite, has not been formally tested. In some cases, trogocytosis results in death of the trogocytosed cell (Matlung et al. 2018; Mercer et al. 2018; Olivera-Valle et al. 2020; Ralston et al. 2014). Human PMNs have recently been observed to use trogocytosis to kill not only *T. vaginalis*, but also cancer cells (Matlung et al. 2018), and sperm cells (Olivera-Valle et al. 2020). A leading hypothesis is that PMNs use trogocytosis in lieu of phagocytosis (whole cell engulfment), when viable targets are too large to engulf whole (Bhakta et al. 2020).

We therefore hypothesize that, similar to human PMN killing of the human-infective trichomonad parasite *T. vaginalis*, bovine PMNs use trogocytosis to kill the bovine-infective trichomonad parasite *T. foetus*. As PMNs are the first responders to infections and are abundant at the infection site during bovine trichomonosis, the knowledge gained in this study will serve as a first step towards a more complete characterization of immune mechanisms at play during bovine trichomonosis, at a cellular and molecular level.

## Materials & methods

### Animals and blood products

Cattle housed at the California State Polytechnic University, Pomona (Cal Poly Pomona) cattle unit were blood donors. The protocol for bovine whole blood collection was approved by the Cal Poly Pomona Institutional Animal Care and Use Committee (protocol 20.031). Whole bovine blood was obtained via jugular venipuncture and collected into 10 mL K_2_EDTA vacutainer tubes (Becton Dickinson) and transported at room temperature. Bovine PMN were then promptly isolated as previously described (Czuprynski and Hamilton 1985; Roth and Kaeberle 1981). Briefly, blood was transferred into conical tubes, and centrifuged at 1000G for 20 min with no brake. The plasma, buffy coat, and ¼ of the top of the red blood cell pellet was aspirated and discarded. ACK lysis buffer (ThermoFisher) was added to the remaining ¾ of the red blood cell pellet and incubated for 15 min, to lyse erythrocytes, followed by an additional 5 min in fresh ACK lysis buffer if needed. Tubes were then centrifuged at 200G for 10 min and washed 3 times with PBS (Gibco). The remaining leukocyte pellet was resuspended in complete bovine RPMI + HEPES media containing 1% Glutamax, 1% pen/strep and 10% heat-inactivated bovine serum (all from Gibco), and passed through a 70uM cell strainer to remove any clumped cells. Functional experiments were commenced promptly. Alternatively, for isolation of PBMCs, blood was diluted 1:2 in HBSS (Gibco) and fractionated using Ficoll-paque PLUS (GE Healthcare) according to the manufacturer’s instructions. The buffy coat was removed and washed with PBS.

For collection of antiserum, one heifer was vaccinated and boosted with TrichGuard™ (Boehringer Ingelheim Vetmedica, Inc.) subcutaneously according to the manufacturer’s instructions. Antiserum was collected as previously described (Liu et al. 2018). Briefly, blood was collected into serum separator tubes (Becton Dickenson), allowed to clot at room temperature for 30 min, and then tubes were spun at 4000G for 10 min in a precooled 4 °C centrifuge. The top layer containing serum was collected and passed through a 0.22 μm filter. Aliquots were stored at -20 °C and thawed gradually on ice prior to use in each functional experiment.

### Annexin V staining

Cells were stained with Annexin V APC (Biolegend) according to the manufacturer’s instructions and analyzed on a MACS quant flow cytometer.

### Parasite strains and cultures

*Tritrichomonas foetus* strain KV-1 (ATCC) was grown in Diamond’s medium (Clark and Diamond 2002). Briefly, TYM medium was supplemented with 10% of heat-inactivated horse serum, and iron solution, and either left untouched (pH6.8), or adjusted to a pH of 6.2 or 7.2. Cultures were maintained at 37^O^C between 0.5 × 10^4^ to 2 × 10^6^ cells/mL, and passaged into fresh media daily in sealed conical tubes. Before passaging or using for experiments, cultures were placed on ice for 15 min to release parasites that had adhered to the plastic conical tube.

### Antibody staining

Isolated leukocytes were stained with anti- bovine GR-1 antibody to confirm PMN identity, as previously described (Etchevers et al. 2023; Lietaer et al. 2021; Lopes et al. 2021; Oliveira et al. 2020). Briefly, cells were stained with mouse anti-bovine granulocyte (GR-1) monoclonal IgM primary antibody (clone-CH138A, Kingfisher Biotech), or isotype control (clone-COLIS52A2, Kingfisher Biotech) at a concentration of 1.0 ug/mL, followed by a FITC goat anti-mouse IgM polyclonal secondary antibody (ThermoFisher) at a concentration of 1.5 ug/mL. Stained cells were then run on a MACSQuant® Analyzer 10 Flow cytometer (Miltenyi Biotec Inc.) and data was analyzed using Flowjo (Becton Dickenson).

### Cytotoxicity assays

Modified flow cytometry-based cytotoxicity assays previously established to evaluate PMN killing of trichomonads (Mercer et al. 2018) were used. For cytotoxicity assays in Figs. 2 and 3, a total of 3 × 10^5^ PMNs and 3 × 10^4^ Cell Tracker Green (CTG) labelled *T. foetus* were co-cultured in RPMI with 10% *T. foetus* antiserum in a 96 well plate at 37° and 5% CO_2_ for the indicated times. Co-cultures were then washed in Annexin V binding buffer (Biolegend) and resuspended in Zombie Violet (Biolegend), at a 1:1000 dilution from the stock, in Annexin V binding buffer and incubated for 30 min at room temperature. Samples were then washed and resuspended in Annexin V binding buffer before running on a MACS Quant 10 flow cytometer (Miltenyi Biotech). Live *T. foetus* (CTG + Zombie Violet- cells) were gated on, as shown in Figure S1A, and percent parasite death was determined using the following calculation: ([ number of live *T. foetus* in negative (parasites only) control – number of *T. foetus* in coculture condition] / number of *T. foetus* in negative control) × 100. Cytotoxicity assays that received trogocytosis inhibitors (Fig. 3) followed the same protocol, but PMNs were pre-incubated with 1.25 ug/mL Cytochalasin D (Tocris) or vehicle (DMSO) or 3.2ug/mL wortmannin (Tocris) or vehicle control (DMSO) for 20 min prior to adding *T. foetus*.

For the alternative cytotoxicity assay (Fig. S3), bovine PMNs were co-cultured with *T. foetus* in complete RPMI containing 10% *T. foetus* antiserum in a 96-well-v-bottom plate for 1 h at 37° in 5% CO_2_. All conditions contained 1.5 × 10^4^ parasites, and the number of PMNs was varied according to the indicated ratios. After incubation, co-cultures were set on ice for 15 min and then centrifugated at 3200 rpm for 10 min at 4 °C, and decanted. Wells were then resuspended in 300uL of pre-warmed complete Diamonds media and left to incubate overnight in sealed plastic bags at 37°. The following day, cocultures were placed on ice for 15 min before reading on a MACS Quant 10 flow cytometer to determine parasite count in each well. Percent killing was calculated as ([ number of *T. foetus* in negative (parasites only) control – number of *T. foetus* in coculture condition] / number of *T. foetus* in negative control) × 100.

### Imaging

To visualize PMNs following isolation, 10ul of isolated cells were used to create a smear on a glass slide and allowed to dry, followed by Leishman staining, in which 8 drops of the stain were added to the slide, left for 2 min, followed by 16 drops of water for 15 min, followed by gentle washing in water. Slides were imaged on a Leica light microscope with the 40 × objective. To visualize trogocytosis, parasites were surface-labelled with EZ-Link Sulfo-NHS-SS-Biotin (ThermoFisher) for 30 min on ice followed by 5ug/mL Streptavidin-Alexa 488 (Biolegend) for 5 min. For 3D imaging, PMNs were pre-labelled with Cell Tracker Red (Molecular Probes) according to the manufacturer’s instructions. A total of 3.6 × 10^6^ PMNs, or Jurkat cells (Fig. S5), were plated in RPMI with 10% *T. foetus* antiserum in a 12-well plate and allowed to settle down on glass coverslips pre-coated with 0.01% poly-L-lysine (MilliporeSigma). Then, 3.6 × 10^5^ labelled *T. foetus* were added in a dropwise manner slowly around the well and incubated for the indicated timepoints. Wells were then gently aspirated, washed twice with PBS and then fixed with 900uL of 4% PFA added gently to the side of the plate to minimize disruption of cellular aggregates. After 10 min, PFA was gently removed, and coverslips were dipped twice in PBS, and once in ddH_2_O before being placed with parasite-PMN aggregates facing down on slides with 20uL of Prolong Gold^©^ anti-fade mounting media (Thermo Fisher). Slides were left flat in the dark to dry overnight before a thin layer of clear coat nail polish was applied around the edges of the coverslip to prevent contamination. Slides were stored at -20 °C and images were obtained using a Nikon Confocal Microscope using the 100 × objective with oil. Images were then analyzed by identifying each intact parasite in the image, and visually counting the number of PMNs touching that parasite, and the number of green fragments localized to PMNs, from each parasite. For 3D imaging, z-stacks were taken every 0.13 um and 3D reconstructions were made using volume viewer in FIJI software.

## Results

### Isolation of PMNs from bovine peripheral blood

**Fig. 1:**
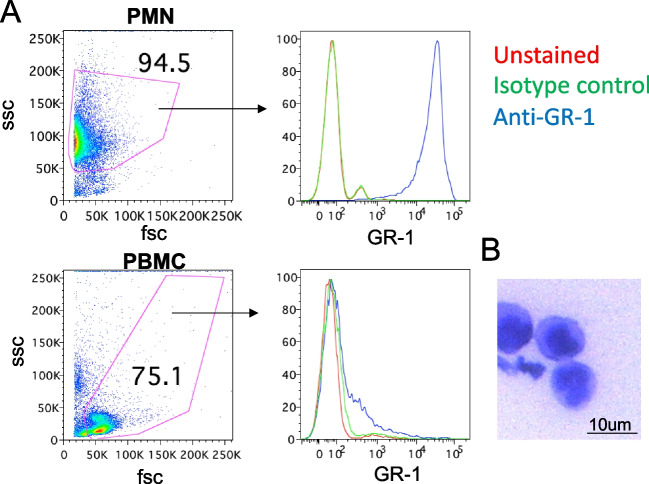
Isolation of PMNs from bovine blood. **A** PMNs or PBMCs were stained with anti-GR-1 antibody and analyzed using Flow Cytometry. PMNs show higher granularity, as assessed by side-scatter (ssc), and higher expression of Granulocyte Receptor-1 (right panels) than PBMCs. Data shown are representative of triplicate samples run in three independent experiments from three different donors. **B** PMN display characteristic multi-lobed nuclei, as visualized using Leishman staining. Image shown is representative of 16 images taken from PMNs isolated from 4 different donors over 2 independent experiments

To test whether and how bovine neutrophils, otherwise known as polymorphonuclear cells (PMN), kill *T. foetus*, we first isolated bovine PMNs from bovine blood. Using previously established centrifugation protocols (Roth and Kaeberle 1981), we isolated Peripheral Blood Mononuclear Cells (PBMC), and PMNs. After staining our fractionated PBMC or PMN cell fractions using an anti- bovine granulocyte monoclonal antibody (GR-1) (Etchevers et al. 2023; Lietaer et al. 2021; Lopes et al. 2021; Oliveira et al. 2020), we found that our PMN fraction was viable, and had a PMN identity, as anti-GR-1 reactivity was very high in all of the PMN cells, but few of the PBMC cells (Fig. 1A). Furthermore, the PMNs displayed a characteristic multi-lobed nuclear morphology (Fig. 1B). As expected, the PMNs were short-lived, as they became apoptotic by the following day (Fig. S1).

### Bovine PMNs rapidly kill *T. foetus* in a dose- dependent manner

**Fig. 2:**
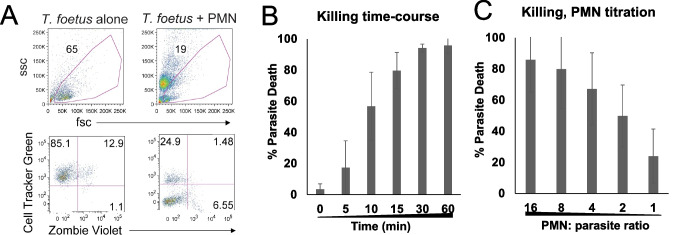
Bovine PMNs rapidly kill *Tritrichomonas foetus* in a dose-dependent manner. (**A**) Representative gating strategy for assessing live parasite using flow cytometry. Cells (upper panel) were gated on, and all gated events were assessed for Cell Tracker Green (CTG) and Zombie violet, to identify live parasite (CTG + , Zombie violet- events). (**B**, **C**) Cytotoxicity assays. Live parasite counts were used to determine percent parasite death, by comparing the amount of live parasites remaining in the co-culture condition to a parasite-alone condition at each timepoint. Bargraphs show the average of three independent experiments with three different donors, all performed in triplicate. Error bars show standard deviations

We next sought to test whether our isolated bovine PMNs were able to kill *T. foetus *in vitro. To test this, we adapted an in vitro cytotoxicity assay previously used to test human PMN killing of the human-infective trichomonad parasite *T. vaginalis* (Mercer et al. 2018). Before the assay, *T. foetus* were pre-labelled with Cell Tracker Green (CTG) to discern them from PMNs following the co-culture. After the co-culture, all cells were spun down, stained with cell-death marker Zombie Violet, and then analyzed using flow cytometry, to identify and count live parasites (CTG + , Zombie Violet- cells) (Fig. 2A). Since a ratio of 10:1 PMN: trichomonad efficiently killed *T. vaginalis* (Mercer et al. 2018), we first started by co-culturing bovine PMNs with *T. foetus* at a 10:1 ratio. To test how rapidly the killing occurred, we analyzed various timepoints, and found that killing occurred rapidly, with low levels of parasite death occurring as early as five minutes, with about half of the parasites dead by ten minutes (Fig. 2B). All of the parasites were killed by 1 h of the co-culture (Fig. 2B). Next, to determine how many PMNs are required for optimal killing of *T. foetus*, we tested bovine PMN killing of *T. foetus* at various PMN: *T. foetus* ratios. Since we found killing to be complete within one hour (Fig. 2B), we used a one-hour timepoint, and set-up co-cultures of *T. foetus* with descending ratios of PMNs (Fig. 2C). We found that only moderate killing of *T. foetus* was observed at PMN: parasite ratios of 1:1 and 1:2, but that as PMN numbers increased, the parasites were killed more efficiently, with about half of the parasites killed at a 4:1 ratio, and very high percentages of parasites killed at 8:1 and 16:1 ratios (Fig. 2C). Therefore, bovine PMNs kill *T. foetus *in vitro rapidly, starting at five minutes, with most killing occurring within fifteen minutes, and completing within one hour. Maximum *T. foetus* killing required multiple neutrophils, with efficient killing requiring at least 4 PMN: 1 parasite, suggesting that bovine PMNs work in aggregates to rapidly attack single trichomonads.

To test an alternative method of assessing PMN killing of parasites, we also wanted to allow the parasites to grow following these co-cultures; any remaining live parasites should expand. Therefore, after determining that the optimal pH of the growth media for *T. foetus* was pH 6.8 (Fig. S2), we co-cultured bovine PMNs and *T. foetus* for one hour to allow killing to proceed, and then spun down the co-cultures and resuspended them in *T. foetus* growth media pH 6.8 for overnight culture (Fig. S3). We then counted parasites the following day and determined the percentage of parasites killed by PMN, by comparing numbers of parasites present in each PMN condition to a *T. foetus* only (no PMN) control. We found the calculated percentages of parasites killed using this alternative method were similar to that seen with the traditional method (Fig. 2C, Fig. S3E). Therefore, we show two valid methods to test parasite killing by PMN in vitro, one with the advantage of being completing in 1 day (Fig. 2), and one that avoids having to pre-label parasites with CTG (Fig. S3), and thus is a cheaper option.

### Bovine neutrophil killing of *T. foetus* is reduced in the presence of trogocytosis inhibitors

**Fig. 3:**
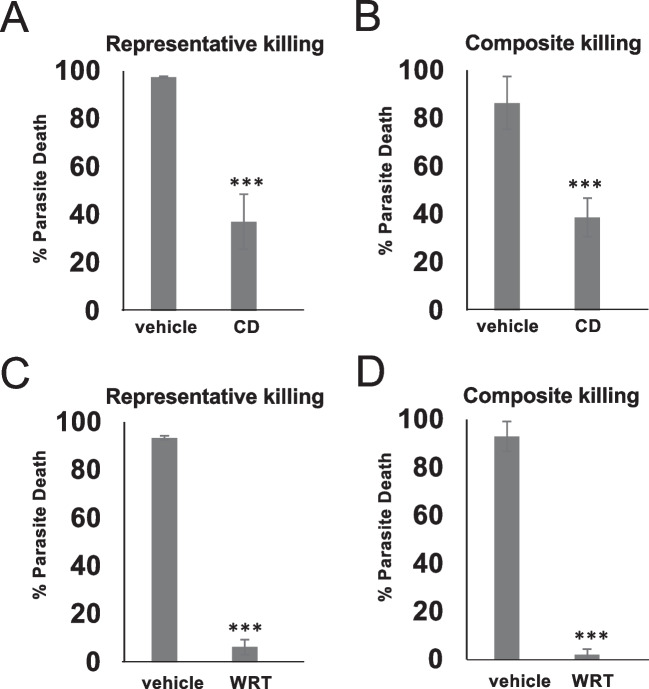
Bovine PMN killing of *Tritrichomonas foetus* is reduced in the presence of trogocytosis inhibitors. Bovine PMNs were pre-incubated with either 1.25 ug/ml of actin polymerization inhibitor Cytochalasin D (CD) (**A**, **B**) or 3.2ug/ml of PI3K inhibitor wortmannin (WRT) (**C**, **D**) for 20 min, before co-culture with cell-tracker green labelled *T. foetus at a ratio of* 16 PMN: 1 parasite for 1 h. Then, cultures were stained with zombie violet to determine surviving cells. Numbers of surviving *T. foetus* (cell tracker green + , zombie violet- cells) were quantified using flow cytometry, and percent killing was calculated by comparing co-culture conditions to a parasite alone control for each condition. Panels A and C show averages of triplicate wells, and panels B and D show composite data from at least 3 independent experiments from at least 3 different blood donors, with triplicate wells each time. Error bars show the standard deviations, and p-values were calculated using a two-tailed T- test

We also observed a rapid killing requiring aggregates of PMNs to attack a single parasite in human PMN killing of the human-infective trichomonad parasite *T. vaginalis*, and found that the PMNs used a novel antimicrobial mechanism termed trogocytosis in this killing (Mercer et al. 2018). We therefore next sought to determine whether bovine PMN killing of *T. foetus* proceeds through trogocytosis. As opposed to phagocytosis, in which PMNs engulf targets whole, trogocytic killing involves PMNs acquiring small fragments of target cells, eventually leading to the target cell’s death (Matlung et al. 2018; Mercer et al. 2018). Trogocytosed material from target cells has been found to localize both to the cell surface (Ochs et al. 2023; Samer et al. 2023; Schriek and Villadangos 2023) as well as inside the trogocytosing cells (Bettadapur et al. 2020; Uribe-Querol and Rosales 2021). To test whether bovine PMNs kill *T. foetus* using trogocytosis, we therefore performed our in vitro cytotoxicity assay as described above (Fig. 2), but first pre-incubated bovine PMNs in two inhibitors: cytochalasin D, which inhibits actin polymerization, and wortmannin, which inhibits PI3K signaling. Both of these inhibitors have previously been shown to inhibit trogocytosis (Aucher et al. 2008; Matlung et al. 2018; Mercer et al. 2018; Olivera-Valle et al. 2020; Pham et al. 2011; Ralston et al. 2014; Shin et al. 2021), including trogocytosis of *T. vaginalis* (Mercer et al. 2018). While neither inhibitor demonstrated any inherent toxicity to the cells (Fig. S4), we found that both inhibitors reduced bovine PMNs killing of *T. foetus*, indicating that PMN trogocytosis of *T. foetus* could be involved in the killing we observed (Fig. 3).

### Bovine neutrophils surround and acquire fragments of *T. foetus’* membrane

Having observed that bovine killing of PMNs occurs rapidly and is reduced in the presence of actin polymerization and PI3K signaling inhibitors, we next sought to determine whether this rapid killing proceeds through phagocytosis (whole target cell engulfment) or trogocytosis (acquisition of fragments of cells), as both processes are inhibited when actin polymerization and PI3K signaling are impaired. Since bovine PMN killing of *T. foetus* was only efficient when PMN: *T. foetus* ratios were higher than 2:1, we hypothesized that killing proceeds through trogocytosis, where multiple PMNs surround one parasite and acquire fragments of it, whereas phagocytosis (whole parasite engulfment by a single PMN) should occur efficiently at a 1:1 PMN: parasite ratio. Furthermore, PMNs killing of *T. vaginalis*, a related trichomonad parasite, proceeds through trogocytosis (Mercer et al. 2018). In addition, *T. foetus’* large size (10-20uM) (Bandeira et al. 2023) compared to bovine PMNs (less than 10uM) (Fingerhut et al. 2020) suggests that whole parasite engulfment by a single PMN is unlikely.

**Fig. 4:**
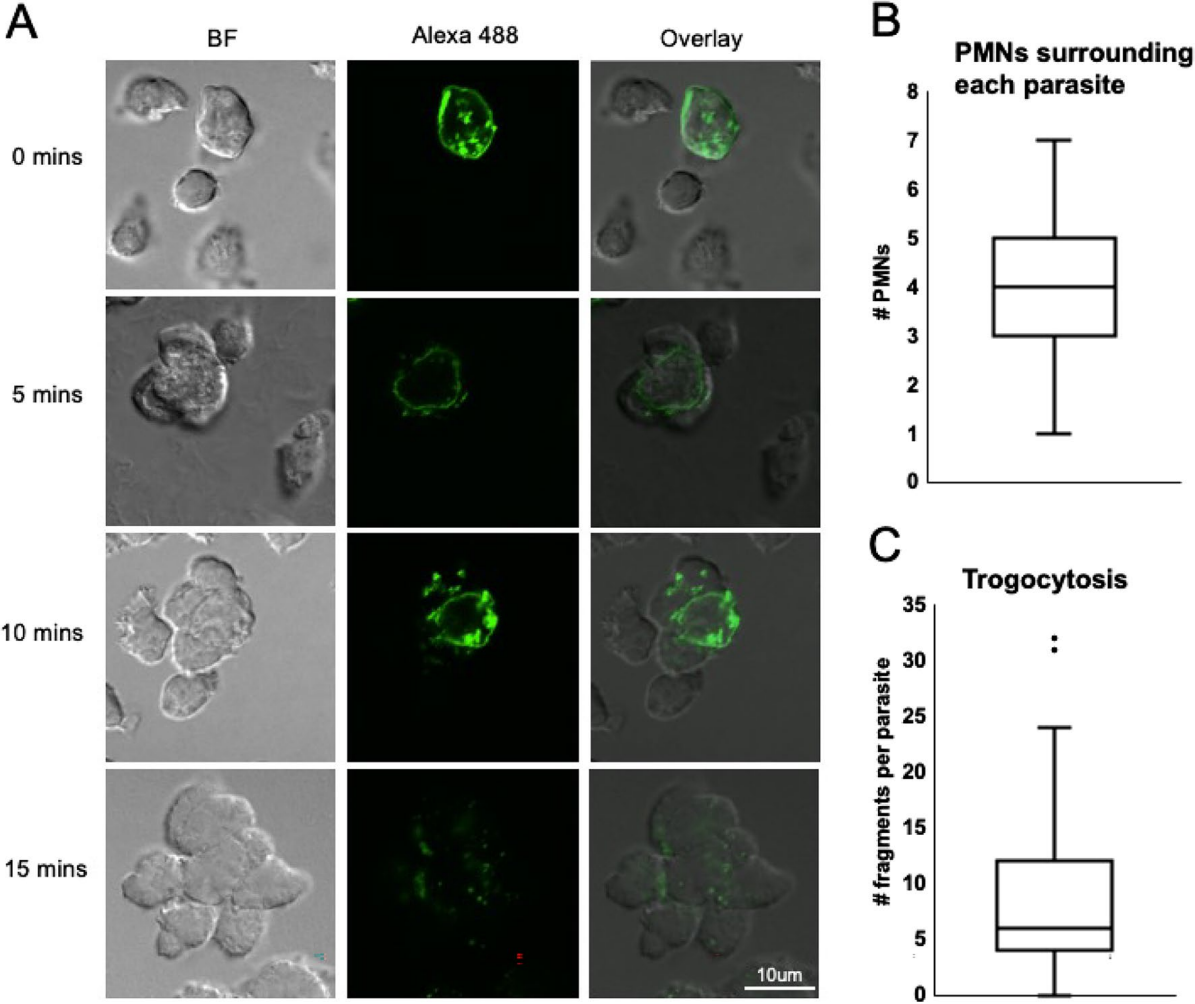
Bovine neutrophils surround and acquire fragments of *Tritrichomonas foetus’* membrane. (**A**) *T. foetus* was surface-labelled (Alexa 488) and added to lawns of bovine PMNs on coverslips in the presence of 10% anti-*T. foetus* antiserum for the indicated timepoints, and then coverslips were fixed and mounted on glass slides for imaging. A representative parasite from an image taken at each timepoint is shown. Altogether, 33 parasites were imaged at time 0, 102 parasites were imaged at 5 min, 55 parasites were imaged at 10 min, and 42 images were taken at 15 min. (**B**, **C**) 102 individual parasites across triplicate coverslips for each of three independent experiments with unique blood donors were visually analyzed for (**B**) the number of PMNs physically touching each individual parasite, and (**C**) the number of green fragments observed to be trogocytosed from each parasite at the 5 min timepoint

**Fig. 5:**
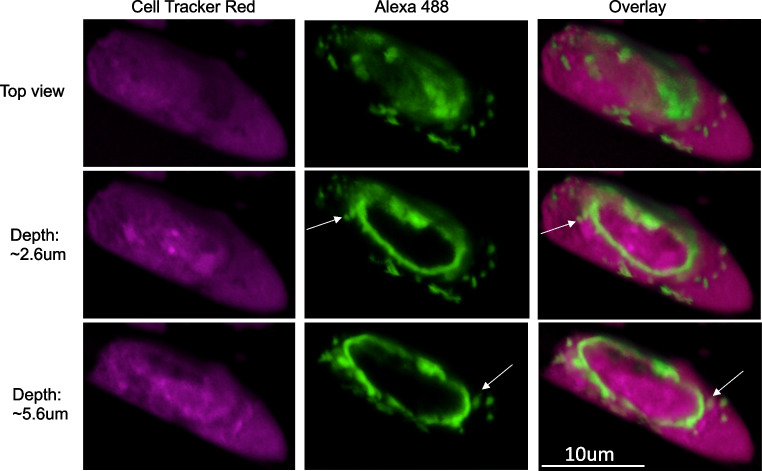
Trogocytosed *T. foetus* membrane fragments localize mostly externally on PMNs. Imaging experiments performed as in Fig. 4, except PMNs were pre-labelled with Cell Tracker Red (red) and samples were imaged as z-stacks using the 100 × objective. The top view, and two cross sections through the image are shown. White arrows indicate *T. foetus* membrane fragments that are located internally in PMNs (not visible from the top view), while fragments located on the PMNs’ surface are left unmarked. The image shown is representative of at least 12 parasites imaged over three independent experiments with triplicate coverslips each

To test whether bovine PMN killing of *T. foetus* proceeds through phagocytosis or trogocytosis, we performed an imaging assay, in which *T. foetus’* plasma membrane was stably labelled, and parasites were added to coverslips containing lawns of bovine PMNs. After allowing the cells to interact for up to fifteen minutes, the timepoint by which a majority of parasites were killed (Fig. 2B), we fixed the coverslips onto slides and imaged them using confocal fluorescent microscopy (Fig. 4). We found that by five minutes, aggregates of PMNs had surrounded individual parasites, and that small fragments of the parasite’s membrane had begun to colocalize with the PMNs. By ten minutes, more fragments accumulated, and by fifteen minutes, we were only able to identify very few intact parasites on our coverslips, but did find many PMNs with green fragments. We never observed transfer of parasite membrane to a control, non-phagocytic cell line (Fig. S5), indicating that the transfer was an active process driven by the PMNs. Higher resolution imaging in 3D revealed that while fragments of *T. foetus’* membrane were found both inside PMNs and on the surface of PMN, a majority of fragments were surface localized (Fig. 5). Therefore, PMNs appear to surround individual parasites and trogocytose them, which leads to parasite death. However, we saw almost no instances of whole parasite engulfment. Similar to human PMN trogocytosis of *T. vaginalis*, we found that while the number of PMNs surrounding individual parasites varied, on average, we observed around four PMNs surrounding each parasite (Fig. 4B).

## Discussion

Despite the serious impacts of *Tritrichomonas foetus* infection on bovine reproduction, control strategies have fallen short of eradicating infection or eliminating disease outcomes. A more complete understanding of how the parasite interacts with the bovine immune system at a cellular and molecular level may potentiate the future design of more robust control or mitigation strategies. As a first step towards characterizing immunity to *T. foetus* at the cellular level, we examined the interaction of *T. foetus* with bovine PMNs, the most abundant immune cells at the site of infection, and the canonical first responder leukocytes. Here, we show that bovine PMNs can rapidly kill *T. foetus *in vitro, in a dose-dependent manner, which occurs efficiently when multiple PMNs can attack a single parasite. Since killing is decreased in the presence of actin polymerization and PI3K signaling inhibitors previously shown to inhibit trogocytosis, and the acquisition of small fragments of *T. foetus’* membrane by PMNs precedes parasite death, these data support a model where bovine PMNs kill *T. foetus* using trogocytosis, a recently described PMN antimicrobial mechanism used against large target cells.

While trogocytosis is not always lethal (Bettadapur et al. 2020; Uribe-Querol and Rosales 2021), trogocytosis as a mode by which PMNs kill targets has been established for target cells including cancer cells (Matlung et al. 2018), sperm cells (Olivera-Valle et al. 2020), and the human-infective trichomonad parasite, *Trichomonas vaginalis* (Mercer et al. 2018). Given the similarities between *T. vaginalis* and *T. foetus* in size and morphology (Vilela and Benchimol 2013), as well as similarities in their host-cell attachment and pathogenesis (Vilela and Benchimol 2013), it is logical that PMNs would combat both parasites similarly.

As we found *T. foetus* to be killed rapidly, with fragments of *T. foetus’* membrane acquired by PMNs preceding parasite death, and with this killing decreased in the presence of trogocytosis inhibitors, we conclude that bovine PMNs use trogocytosis at least in part, to kill *T. foetus*, similarly to how human PMNs use trogocytosis to kill *T. vaginalis*. We found several similarities in PMN killing of both *T. foetus* and *T. vaginalis*, such as the timing, and the ratios at which the killing occurred most efficiently. Killing of both *T. foetus* and *T. vaginalis* was rapid, with substantial parasite death occurring as early as 5–10 min following PMN- trichomonad contact (Fig. 2B) (Mercer et al. 2018), and with substantial killing requiring aggregates of PMN to surround parasites; ratios of at least four PMN:one trichomonad were required to kill greater than 50% of the trichomonads present (Fig. 2C) (Mercer et al. 2018).

PMNs have three other established mechanisms by which they kill targets: phagocytosis (whole-cell engulfment), extracellular degranulation (exocytosis of toxic granules), and the casting of Neutrophil Extracellular Traps (NETosis), in which pathogens become ensnared in a web of chromatin released extracellularly from the PMN nucleus (Kolaczkowska and Kubes 2013). While NETosis may also be useful against large targets that are too big to engulf whole, it is generally viewed as a suicide mission, employed as a last-ditch effort when other modes have failed; NETs are released in vitro after 2–4 h of stimulation (Mercer et al. 2018; Ramirez-Ledesma et al. 2022). In contrast, the trichomonad killing we observed both here, and in our previous studies, occurred rapidly, with trogocytosis observed almost immediately following PMN-trichomonad contact (Figs. 2B and 4) (Mercer et al. 2018), and a majority of trichomonad death following within the next 10–15 min (Figs. 2B and 4) (Mercer et al. 2018). Furthermore, in our previous studies, we ruled out the role of NETosis in PMNs killing of *T. vaginalis* by performing cytotoxicity assays in the presence of DNAse that degraded PMN NETs, and saw no effect on killing within a two-hour timeframe (Mercer et al. 2018). However, it is possible that NETosis may play a role in killing of trichomonads at later time-points, for those parasites that were resistant to the initial trogocytic attempts, or under conditions that are unfavorable for trogocytosis, such as when parasites are highly clumped and therefore inaccessible to direct PMN contact, or in the absence of opsonins (Bhakta et al. 2020; Ramirez-Ledesma et al. 2022).

Of the remaining two PMN antimicrobial mechanisms, phagocytosis can be ruled out as a trichomonad killing mechanism utilized by PMNs because visually, whole parasite engulfment is not observed (Fig. 4) (Mercer et al. 2018); however, the role of extracellular degranulation is still in question. In our previous studies, we showed that exocytosis of PMN toxic granules into the extracellular space is not toxic to *T. vaginalis*, because parasite death only occurred when the PMNs and parasites came into contact; separation of parasites and PMNs in separate chambers of a transwell plate sharing continuous media prevented killing, even when PMNs were stimulated with a strong agonist to induce degranulation (Mercer et al. 2018). However, it is not known whether the exocytosis of PMN toxic granules directly onto the trichomonas surface when the cells are in direct contact could contribute to degradation of the parasite’s plasma membrane into fragments. In fact, we and others identified PMN serine proteases as a potential driver of trogocytosis (Mercer et al. 2018; Olivera-Valle et al. 2020), and many PMN serine proteases are present in PMN toxic granules (Kettritz 2016; Pham 2006). Furthermore, studies imaging the interaction between *T. foetus* and rat neutrophils showed that neutrophil granule contents were observed adjacent to the parasite following contact (De Azevedo and De Souza 1993). Therefore, while phagocytosis and NETosis can be ruled out as killing mechanisms that bovine PMNs in this rapid killing of *T. foetus*, the role of PMN extracellular degranulation either independently of, or in conjunction with trogocytosis, should still be investigated.

Furthermore, the subcellular localization of trogocytosed parasite material and downstream immunological consequences of PMN killing of targets using trogocytosis are yet unknown. Some instances of trogocytosis merely involve display of plasma membrane proteins from the trogocytosed cell to the recipient cell’s membrane (Daubeuf et al. 2010; Joly and Hudrisier 2003), while other instances have demonstrated trogocytosed fragments internalized into the recipient cell (Mercer et al. 2018; Ralston et al. 2014). When macrophages engage in trogocytosis, the engulfed material ends up in a double-membraned vesicle inside the recipient macrophage (Steele et al. 2016, 2019), indicating that the trogocytosed material may enter the endocytic pathway rather than directly enter the cytosol. In trogocytosis by *E. histolytica*, sustained trogocytosis and target killing requires lysosomal digestion of the initial bites (Gilmartin et al. 2017), suggesting a role for the lysosome in processing trogocytosed material. Here, we report that *T. foetus’* membrane was found both internally, and on the surface of PMNs (Fig. 5), however it is not yet- known which specific subcellular pathways the trogocytosed material follows to arrive at these locations. Nonetheless, the substantial amount of trogocytosed material that localizes to the PMN surface suggests a role for plasma membrane display of parasite antigen in downstream immune responses (Herbst et al. 2022).

PMN are generally short-lived cells, however, macrophages that efferocytose (Bratton and Henson 2011; Martin et al. 2014) dead PMN-parasite complexes and rare populations of long-lived PMNs (Shim et al. 2022) may play a role in presenting trogocytosed material to T-cells. Therefore, future studies to determine which downstream immune responses are triggered following PMN trogocytic killing of trichomonads, and why, are warranted.

Bovine trichomonosis is a stubborn and persistent problem in cattle, with deleterious effects on reproduction. Improved understanding of cellular and molecular immune mechanisms against the parasite may help to shed light on why infection results in infertility and abortion, and why natural immunity and immunity induced by the existing vaccines is weak and short-lived. As PMN are both the major immune cells present in infected tissues, and play major roles in mediating inflammation, understanding how they interact with the parasite is an important first step towards delineating mechanisms of parasite clearance, persistence, and collateral damage. Understanding the interaction of immune cells with *T. foetus* in sufficient molecular detail may inform intervention strategies that can prolong immunity and/or minimize pathology. Furthermore, trogocytosis is a novel antimicrobial mechanism, and this study expands the range of targets seen to be killed by PMNs using trogocytosis, pointing to the importance of trogocytosis as a fundamental cell process to further explore in its molecular mechanisms, downstream effects, and broader impacts on host–pathogen interactions.

### Supplementary Information

Below is the link to the electronic supplementary material.

Supplementary file1 (PDF 1117 KB)
