# Supplementary material for: Bovine neutrophils kill the sexually-transmitted parasite Tritrichomonas foetus using trogocytosis
Source: Vet Res Commun. 2023 Nov 16;48(2):865–75. doi: 10.1007/s11259-023-10260-5 (PMC10998815; doi:10.1007/s11259-023-10260-5)
Supplement: Supplementary file 1 — Supplementary file1 (PDF 1117 KB) [file 11259_2023_10260_MOESM1_ESM.pdf]

Figure S1

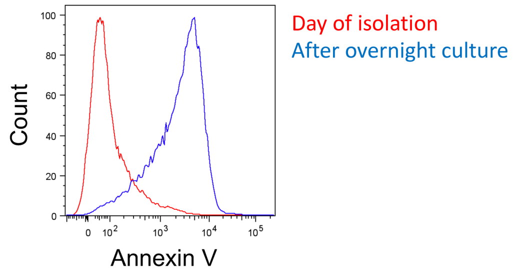

Figure S1: Bovine PMNs are short-lived

Bovine neutrophils were stained with AnnexinV, an indicator of apoptosis, on the same day of isolation, or after an overnight culture in complete RPMI media. All PMN become apoptotic after overnight culture.

Figure S2

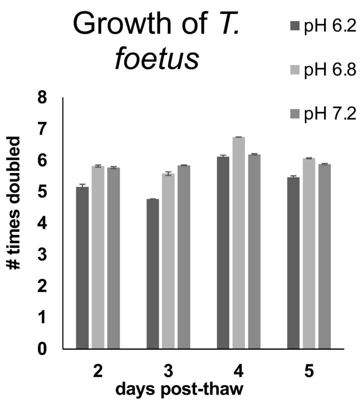

Figure S2: *T. foetus* strain KV-1 grows optimally at pH 6.8

T. foetus was thawed and passaged daily in complete Diamonds media prepared at the indicated pH. Every 24 hours, an equal volume of each culture was analyzed using flow cytometry to determine the parasite count per mL, allowing calculation of how many times each culture had doubled since the previous day. Data shown are average of triplicate wells and representative of two independent experiments. Error bars show the standard deviation.

Figure S3

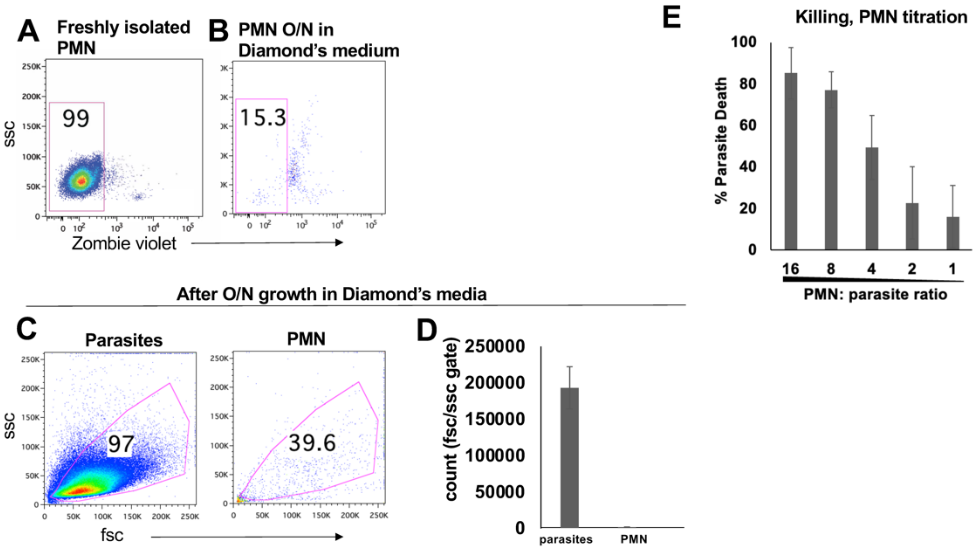

Figure S3: Alternative cytotoxicity assay using standard microbiological growth assay

(A,B) Bovine PMN are not viable after overnight growth in Diamonds media. (A) Bovine PMN were isolated (Figure 1) and then stained with Zombie violet viability kit. The percent of Zombie violet – cells (live cells) within the fcs/ ssc gate from Figure 1 is shown. Data are representative of triplicate samples from two donors. (B) Bovine PMN were cultured overnight in Diamonds growth media under anaerobic conditions, and then stained with Zombie violet. The percent Zombie violet – cells (live cells) within the fsc/ssc gate is shown. Data are representative of triplicate samples from four independent experiments. (C,D) Flow Cytometry to count live cells only detects parasites after an overnight culture in Diamonds media. (C) A representative fcs/ssc gate used for counting parasites after overnight growth in Diamonds growth media is shown. Data are representative of triplicate samples from 6 independent experiments (D) The number of events counted in the fcs/ssc gate shown in (C) is shown. Data are average of at least 6 replicates for each of at least two independent experiments and error bars show standard deviation. (E) The number of parasites the grew up overnight after a 1-hour co-culture with PMN at the indicated ratios. Data re representative of at least three inedpendant experiments with four different donors, each containing triplicate samples. Error bars show the standard deviation.

Figure S4

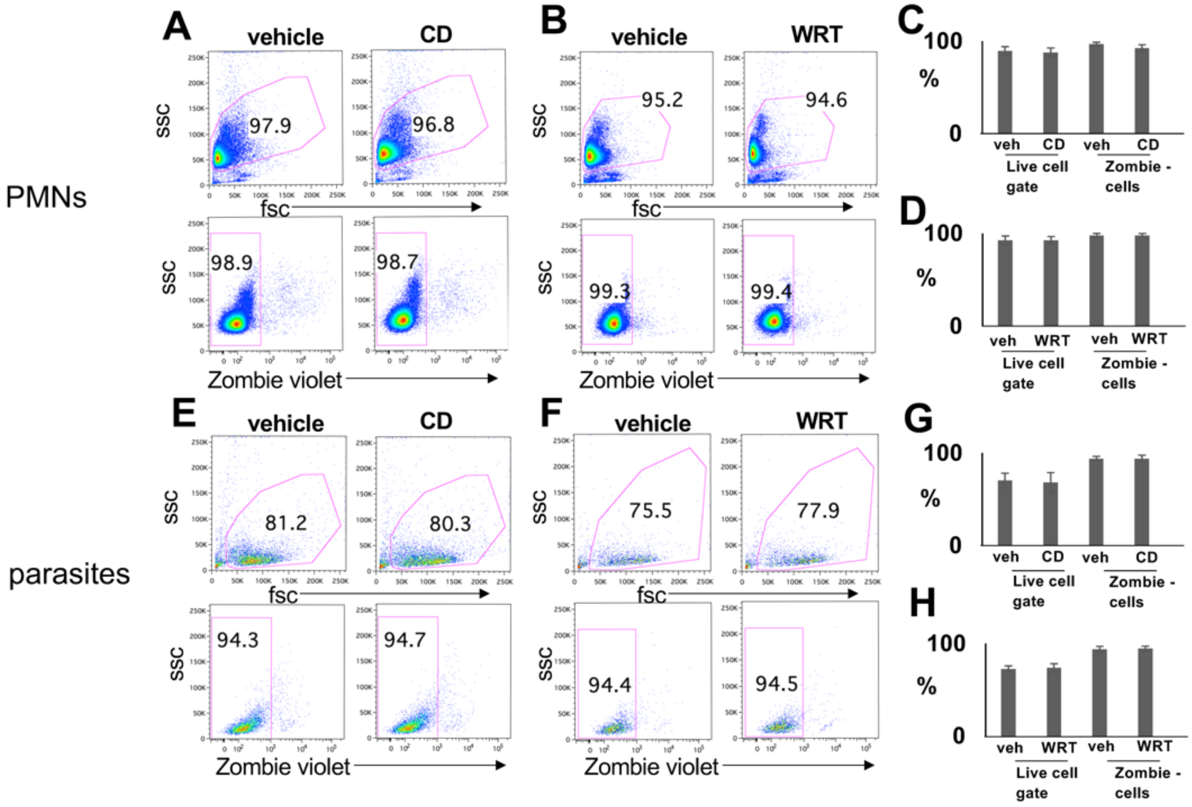

Figure S4: Trogocytosis inhibitors do not affect bovine PMN and *T. foetus* viability

(A,B) Bovine PMN were incubated in 1.25ug/ml Cytochalasin D (CD), or 3.2 ug/ml wortmannin (WRT) or an equivalent volume of DMSO (vehicle) for 90 minutes, followed by zombie violet staining and analysis using flow cytometry. Representative plots of live cell gate (forward-scatter vs side-scatter) and the percentage of cells in the live cell gate that are negative for zombie violet staining are shown. (C,D) Analysis done in A and B was performed using PMN from at least 3 different donors in at least 3 independent experiments in triplicate each time. The average percentage of cells in the indicated population shown. Error bars show standard deviation. (E,F) *T. foetus* were incubated with 1.25ug/ml Cytochalasin D (CD) or 3.2 ug/ml wortmannin (WRT) for 1 hour, followed by zombie violet staining and analysis using flow cytometry. Representative plots of live cell gate (forward-scatter vs side-scatter) and the percentage of cells in the live cell gate that are negative for zombie violet staining are shown. (G,H) Analysis done in E and F was performed in 3 independent experiments in triplicate each time. The average percentage of cells in the indicated population shown. Error bars show standard deviation.

Figure S5

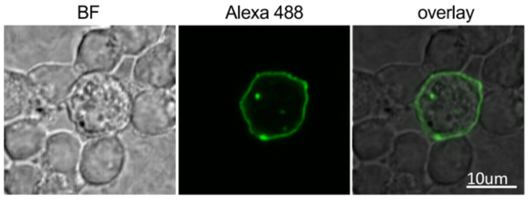

Figure S5: *T. foetus* membrane material is not passively acquired by nonphagocytic cells.

(A) *T. foetus* was surface-labelled (Alexa 488) and added to pre-plated Jurkat cells on coverslips for 15 minutes, and then coverslips were fixed and mounted on glass slides for imaging. A representative parasite image is shown from three independent experiments done with at least duplicate coverslips each time, and at least 3 images from each coverslip.
